# Chloroquine Inhibits Stemness of Esophageal Squamous Cell Carcinoma Cells Through Targeting CXCR4-STAT3 Pathway

**DOI:** 10.3389/fonc.2020.00311

**Published:** 2020-03-13

**Authors:** Dongli Yue, Daiqun Zhang, Xiaojuan Shi, Shasha Liu, Anqi Li, Dong Wang, Guohui Qin, Yu Ping, Yamin Qiao, Xinfeng Chen, Feng Wang, Renyin Chen, Song Zhao, Lidong Wang, Yi Zhang

**Affiliations:** ^1^Biotherapy Center, The First Affiliated Hospital of Zhengzhou University, Zhengzhou, China; ^2^Cancer Center, The First Affiliated Hospital of Zhengzhou University, Zhengzhou, China; ^3^Department of Pathology, The First Affiliated Hospital of Zhengzhou University, Zhengzhou, China; ^4^Department of Thoracic Surgery, The First Affiliated Hospital of Zhengzhou University, Zhengzhou, China; ^5^Henan Key Laboratory for Esophageal Cancer Research and State Key Laboratory for Esophageal Cancer Prevention & Treatment of The First Affiliated Hospital of Zhengzhou University, Zhengzhou, China; ^6^School of Life Sciences, Zhengzhou University, Zhengzhou, China; ^7^Henan Key Laboratory for Tumor Immunology and Biotherapy, Zhengzhou, China

**Keywords:** esophageal squamous cell carcinoma (ESCC), cancer stem cells (CSCs), CXCR4, chloroquine (CQ), STAT3

## Abstract

Esophageal squamous cell carcinoma (ESCC) is one of the most prevalent cancers worldwide. Recent studies have shown that cancer stem cells (CSCs) are present in ESCC, are thought to lead to aggressive tumor behavior and the prognosis. The CXC chemokine receptor 4 (CXCR4), is regarded as a putative CSCs marker in various malignancies. Here, we demonstrate that CXCR4 played a key role in ESCC progression and CXCR4 positive ESCC cells possessed stem-like properties. Furthermore, the anti-malarial agent chloroquine (CQ) targeted CXCR4-positive ESCC cells via STAT3 pathway. Therefore, CQ with anti-CSCs effects may be an effective adjunct to current ESCC chemotherapy regimens.

## Introduction

Esophageal cancer is the fifth and eighth most frequent cause of cancer-related death in male and female worldwide, respectively (1). Esophageal squamous cell carcinoma (ESCC) is the predominant histopathological subtype and comprises up to 90% of esophageal cancer cases worldwide. The incidence of ESCC varies widely by region. The highest prevalence of ESCC was reported in Southern and Eastern Africa and Eastern Asia, particularly in Linzhou of China, however, in Western and Middle Africa and Central America, the lowest prevalence was estimated (2). The prognosis for ESCC remains poor largely due to late diagnosis and propensity for metastasis. Despite significant advances in diagnostic techniques and therapeutic approaches, the overall 5-year survival rate ranges from 15 to 25% (3–5). Recent studies have shown that many malignancies contain a cell subpopulation known as cancer stem cells (CSCs), which are thought to cause aggressive tumor behavior and therapy resistance and have high tumor-initiating capacity, thereby leading to recurrence, metastasis, and insufficient response to conventional therapies (6). Therefore, CSCs markers that are enriched in ESCC must be determined to isolate cells with stem-like characteristics, improving the prognosis of ESCC patients.

The CXC chemokine receptor 4 (CXCR4), a G-protein-coupled receptor (GPCR), also known as a co-receptor for HIV-1 and HIV-2 (7, 8), is activated exclusively by chemokine CXCL12 (9). Although initial studies were centered on the participation of CXCR4 in HIV infection of T cells, its connection to cancer became a hot research topic. CXCR4 was overexpressed in many types of human cancers, and its expression was correlated with angiogenesis, metastasis, and prognosis (10–14). Cancer cells are thought to hijack the CXCR4/CXCL12 axis to establish distant metastasis. The abrogation of the CXCR4/CXCL12 axis results in reduced metastatic burden in a variety of mouse models of cancer. Increasing evidence suggested that CXCR4 could be regarded as a putative CSCs marker in various malignancies, including glioma, non-small cell lung cancer, prostate cancer, renal cell carcinoma, and breast cancer (15, 16). CXCR4 expression in CSCs confers increased invasiveness and metastatic potential as well as improved self-renewal and survival capacity (17). The anti-malarial agent chloroquine (CQ) has been suggested to have an impact on the cancer stem-like cells via inhibition of autophagy (18, 19). Recently, It has been reported that CQ might be a novel antagonist to CXCR4 (20). Balic et al. demonstrated that CQ preferentially targeted CSCs via inhibition of CXCR4 and hedgehog signaling in pancreatic cancer, which acted through autophagy-independent mode of action (21).

In this study, we confirmed that CXCR4 was significantly up-regulated in ESCC specimens and overall survival was significantly shorter for patients with CXCR4 overexpression, suggesting that CXCR4 play a key role in ESCC progression. CXCR4^+^ ESCC cells exhibited high expression of stemness-related genes, had higher metastatic ability, and more resistant to anti-cancer drugs, indicating that CXCR4^+^ cells may possess some characteristics of CSCs. Furthermore, CQ targeted CXCR4-positive ESCC cells via STAT3 pathway, independent of autophagy.

## Materials and Methods

### Cell Lines and Cell Culture

The human ESCC cell line (EC109) were maintained in RPMI 1640 medium containing 10% fetal bovine serum supplemented with 100 U/ml penicillin G and 100 μg/ml streptomycin in humidified atmosphere at 37°C, 5% CO_2_.

### Tissue Specimens and Clinicopathological Characteristics

A total of 121 ESCC tissues and 91 neighboring non-cancerous tissues were collected immediately following surgical resection of ESCC patients at the First Affiliated Hospital of Zhengzhou University (Zhengzhou, China). Ethical approval from the First Affiliated Hospital of Zhengzhou University Research Ethics Committee and patient written informed consent were obtained. Histological examination was carried out by pathologists. None of the patients received any anti-tumor therapy prior to the surgical resection.

### RNA Isolation, Reverse Transcription, and Quantitative Reverse Transcription Polymerase Chain Reaction (qRT-PCR)

Total RNA was isolated using RNAiso Plus (Takara, Japan) and reverse-transcribed using PrimeScript™ II 1st Strand cDNA Synthesis Kit (Takara, Japan) according to the manufacturer's instructions as described earlier (22). QRT-PCR was performed on Agilent Mx3005P system using FastStart Essential DNA Green Master (Roche, USA). Human GAPDH were used as an internal control. All reactions were performed in triplicate. The data were analyzed by 2^ΔΔCt^ method. All the primers used in this research are in Table 1.

**Table 1:** Primer sequences are shown for all genes tested.

**Gene name**	**Sense sequence**	**Anti-sense sequence**	**Product size (bp)**
GAPDH	GCACCGTCAAGGCTGAGAAC	TGGTGAAGACGCCAGTGGA	138
CXCR4	ACTACACCGAGGAAATGGGCT	CCCACAATGCCAGTTAAGAAGA	133
OCT4	CCCCTGGTGCCGTGAAG	GCAAATTGCTCGAGTTCTTTCTG	97
NANOG	CAAAGGCAAACAACCCACTT	TCTGCTGGAGGCTGAGGTAT	158
SOX9	AGCGAACGCACATCAAGAC	CTGTAGGCGATCTGTTGGGG	85
LIN28	CGGGCATCTGTAAGTGGTTC	CAGACCCTTGGCTGACTTCT	191
MMP9	TGTACCGCTATGGTTACACTCG	GGCAGGGACAGTTGCTTCT	97
MMP15	AGGTCCATGCCGAGAACTG	GTCTCTTCGTCGAGCACACC	156
ATG7	ATGATCCCTGTAACTTAGCCCA	CACGGAAGCAAACAACTTCAAC	114
BECN1	CCATGCAGGTGAGCTTCGT	GAATCTGCGAGAGACACCATC	215

### Transwell Migration and Invasion Assay

The migration and invasion of ESCC cells were examine using 24-well Transwell chambers (8-μm pore size polycarbonate membrane, Corning, USA). Harvested cells (2 × 10^4^) were resuspended in 100 μl serum-free RMPI-1640 medium and placed in the upper compartment of the chamber. A total of 500 μl of RMPI-1640 medium containing 10% fetal bovine serum was used as a source of chemoattractant and was added in the bottom compartment of the chamber. After 24 h, the cells that had migrated to the basal side of the membrane were stained with crystal violet (0.005%, sigma, USA) and quantified by counting 5 independent symmetrical visual fields under the microscope. All experiments were repeated three times independently.

### Immunohistochemistry

Formalin-fixed, paraffin-embedded sections (3 mm) were deparaffinized in xylene, rehydrated with an alcohol gradient, and washed briefly in tap water. Endogenous peroxidase was blocked by with methanol containing 0.3% hydrogen peroxide (H_2_O_2_) for 30 min. To retrieve antigenicity, sections were boiled in 10 mM citrate buffer (pH 5.8) for 30 min in a microwave. Next, sections were incubated with goat serum diluted in PBS (pH 7.4) for 30 min at 22°C. Subsequently, sections were incubated at 4°C overnight with primary antibody specific for CXCR4 diluted at 1:200 (Abcam, Cambridge, UK). After incubation with horseradish peroxidase-conjugated secondary antibody for 1 h at 37°C, sections were treated with substrate 3,3-Diaminobenzidine (DAB) and counterstained with hematoxylin and visualized under a microscope (Leica, USA).

### Separation of CXCR4^+^ Cells by Magnetic Cell Sorting (MACS)

EC109 cells were harvested, prepared into single cell suspension and counted. CXCR4^+^ cells were separated according to the manufacturer's instructions. <2 × 10^7^ cells were suspended in 200 μl PEB buffer. Twenty microliter of CD184 (CXCR4)-APC was added, mixed and incubated for 10 min in the refrigerator (2–8°C). The cells were washed and resuspended in 160 μl PEB buffer. Forty microliter of Anti-APC MicroBeads was added, mixed well and incubated for 15 min in the refrigerator (2–8°C). Then cells were washed, resuspended in 1,000 μl PB buffer and proceeded to magnetic separation. The flow-through was unlabeled cells (CXCR4^−^ cells). MACS column was removed from MACS separator and the magnetically labeled cells (CXCR^+^ cells) were obtained.

### Mouse Experiment

Animal protocols were approved by the Animal Care and Use Committee of the First Affiliated Hospital of Zhengzhou University. Female BALB/c-nu mice (15–18 g, 5–6 weeks old) were obtained from the animal facility (Beijing Vital River Laboratory Animal Technology Co., Ltd.). ESCC cells (1 × 10^6^) were injected subcutaneously into the left flank of recipient BALB/c-nu mice. Tumor bearing mice were treated with CQ (50 mg/kg, once daily I.P. for 18 days). The control mice received vehicle (PBS) alone. Tumor growth was monitored using a caliper and tumor volume was calculated according to the formula: (L × W × W)/2.

### Statistics

Statistical analyses were performed with SPSS 17.0. All data were presented as means ± SD of experiments performed in triplicate. Data were tested for normal distribution and Student's *t*-test, ANOVAs, or non-parametric Mann-Whitney U/Wilcoxon tests were applied. A *P* < 0.05 was accepted as statistically significant.

## Results

### CXCR4 Expression Was Correlated With Tumor Invasion and Survival

**Figure 1:**
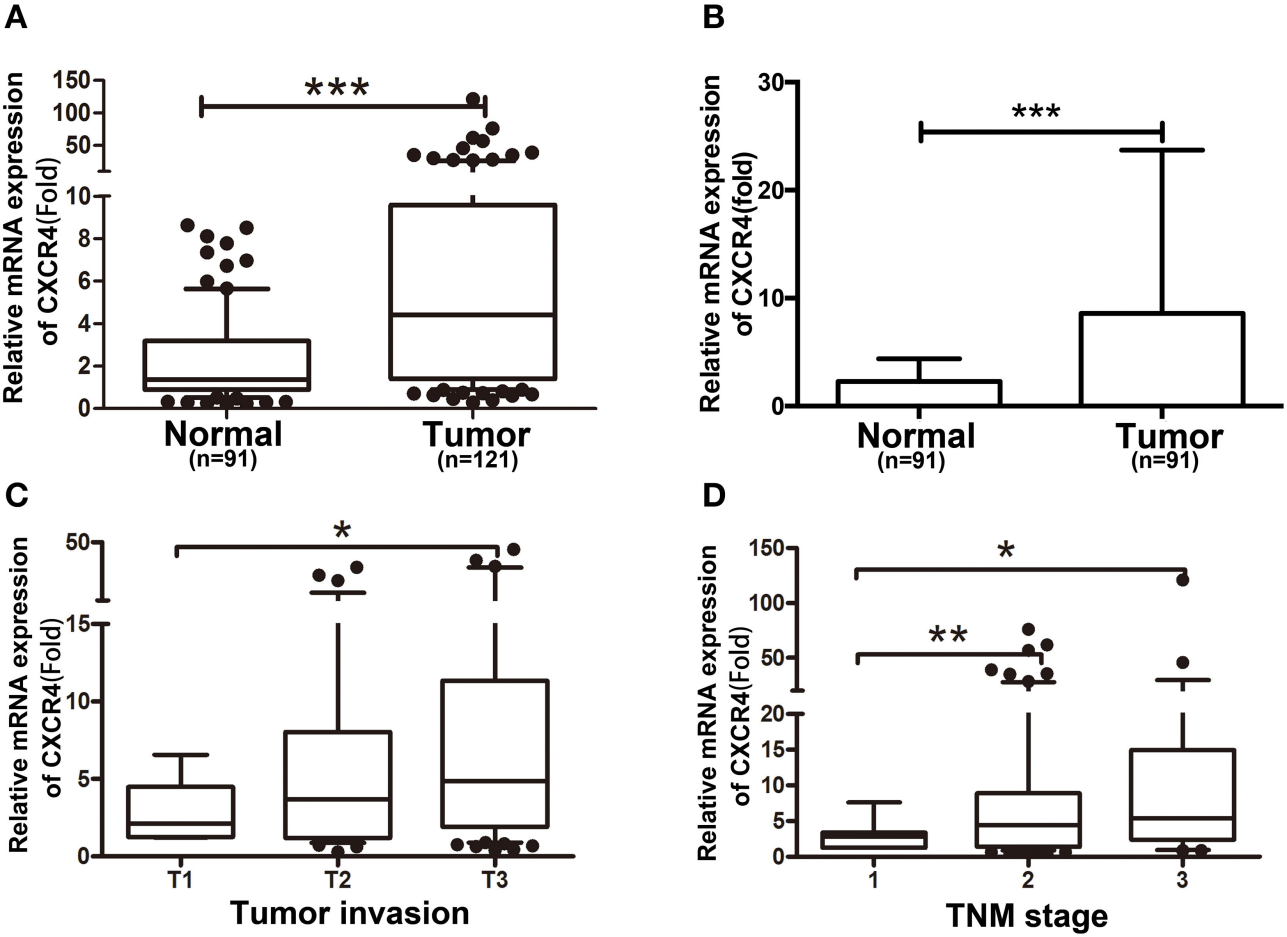
CXCR4 expression is increased in ESCC tissues. **(A)** The expression of CXCR4 in neighboring non-cancerous tissues (Normal, *n* = 91) and ESCC tissues (Tumor, *n* = 121) was determined by real time PCR. **(B)** Expression of CXCR4 in 91 representative carcinoma tissues and their corresponding non-cancerous tissues from the same patients were analyzed side by side for comparison. The correlation of NEDD9 expression with clinicopathological characteristics such as tumor invasion **(C)** and TNM stage **(D)** was analyzed. *P < 0.05, **P < 0.01, and ***P < 0.001.

**Figure 2:**
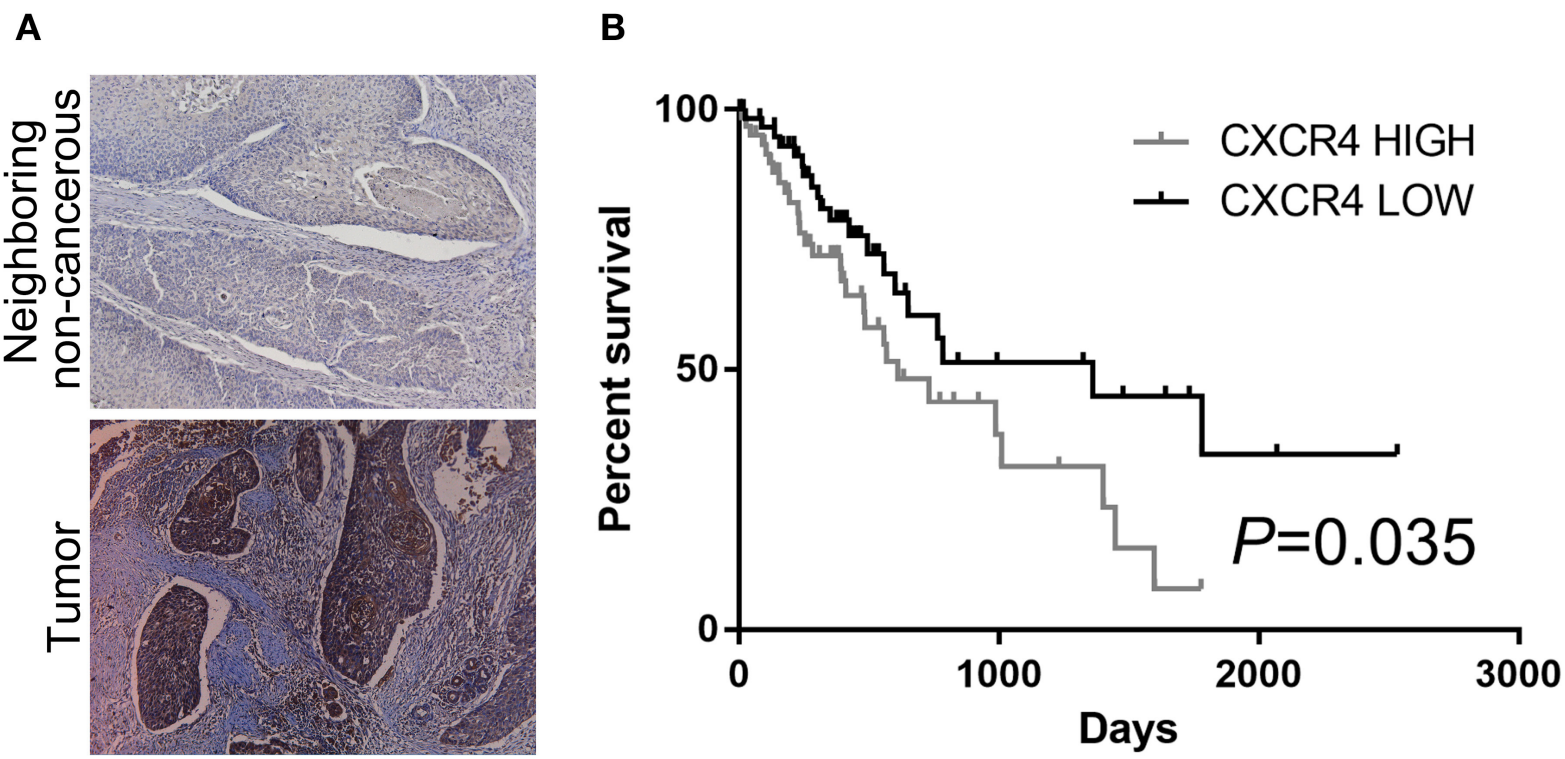
Kaplan-Meier survival analysis in patients with ESCC. **(A)** Representative immunohistochemical staining of CXCR4 expression in ESCC tissues and their corresponding non-cancerous tissues (magnification, ×100). **(B)** Kaplan-Meier analysis indicating the correlation of CXCR4 overexpression with poorer overall survival of ESCC patients.

We first detected CXCR4 mRNA expression in human ESCC specimens and neighboring non-cancerous specimens. We found CXCR4 was significantly up-regulated in ESCC specimens, compared with non-cancerous specimens (Figure 1A). Further, 91 paired ESCC specimens and the corresponding neighboring non-cancerous specimens were analyzed, showing significant up-regulation of CXCR4 expression in ESCC specimens (Figure 1B). Moreover, CXCR4 expression was significantly correlated with tumor invasion and stage (Figures 1C,D). No correlation was observed between CXCR4 expression and patient's age, sex, lymph node metastasis and differentiation. Then we performed immunohistochemistry (IHC) on a paraffin-embedded human ESCC tissues. We found that CXCR4 protein expression was significantly upregulated in ESCC compared with the adjacent non-cancerous tissues (Figure 2A). Further, Kaplan-Meier analysis showed that overall survival was significantly shorter for patients with CXCR4 overexpression (Figure 2B). These data suggested that CXCR4 might play an oncogenic role in human ESCC tumorigenesis and development.

### CXCR4 Positive ESCC Cells Possessed Stem-Like Properties

**Figure 3:**
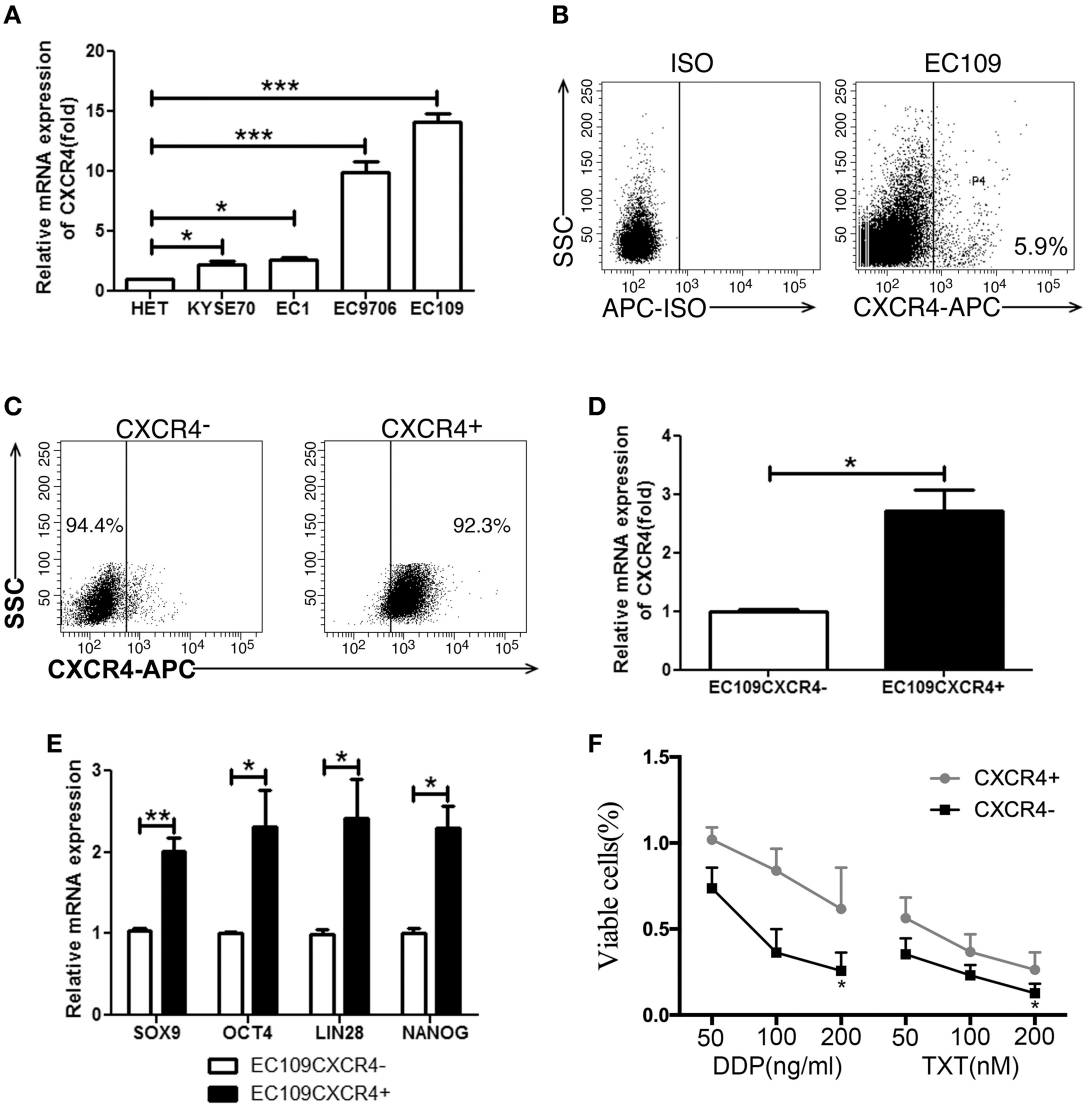
CXCR4 positive cells possessed stem-like properties. **(A)** The CXCR4 expression was detected by real time PCR in 1 immortalized esophageal cell line (Het-1a) and 4 ESCC cell lines. **(B)** The expression analysis of CXCR4 was detected by flow cytometry. **(C)** The purity of sorted EC109 cells with or without CXCR4 expression. **(D)** The expression analysis of CXCR4 was detected by real time PCR. **(E)** The expression analysis of stemness-related transcription factors (SOX9, OCT4, LIN28, and NANOG) was detected by real time PCR. **(F)** Cell survival rate was tested by CCK-8 method. *P < 0.05, **P < 0.01, and ***P < 0.001.

**Figure 4:**
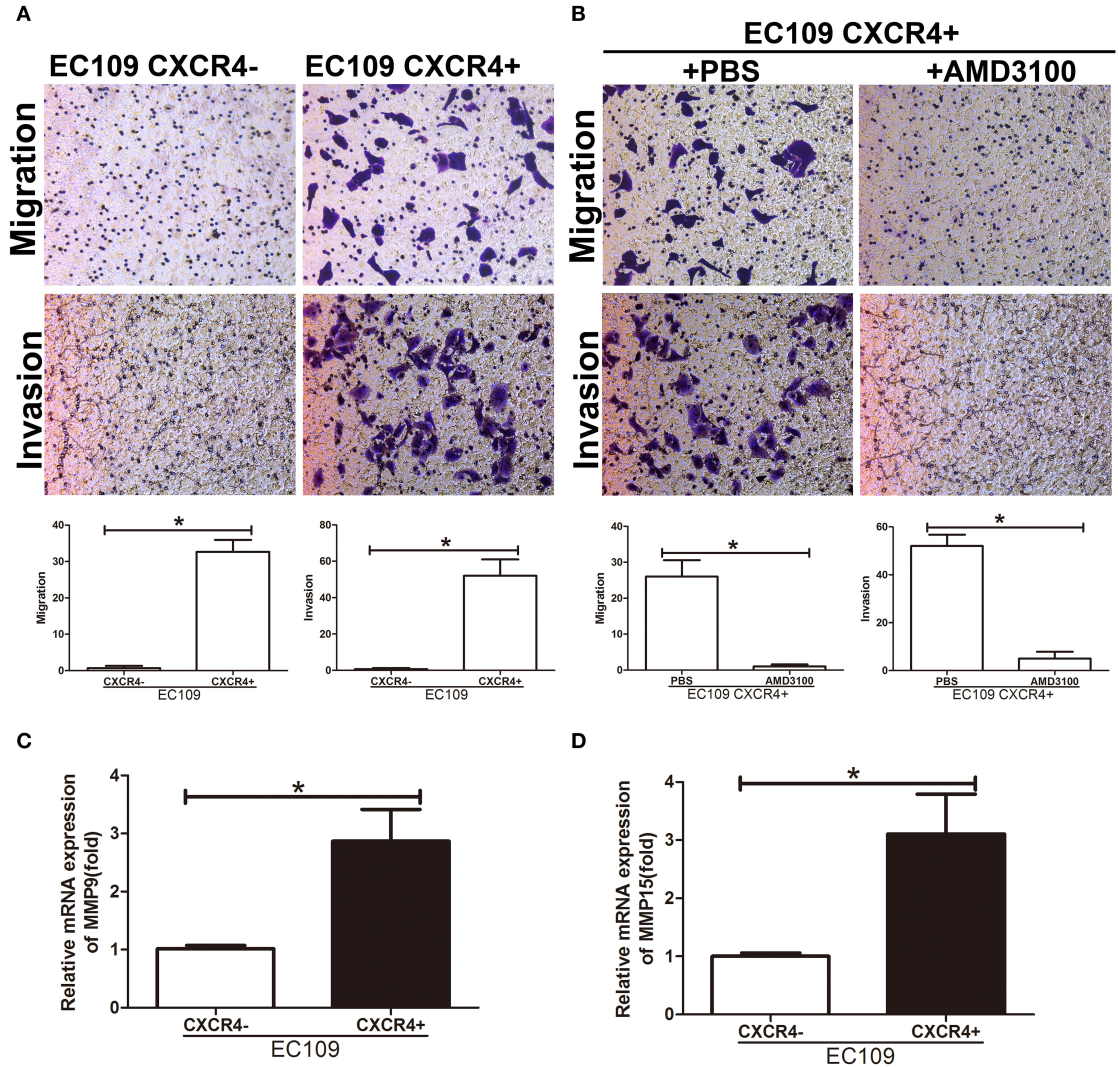
CXCR4 promoted cell migration and invasion. **(A,B)** Migration and invasion assays were performed using Transwell chambers. **(C)** The expression analysis of MMP9 was detected by real time PCR. **(D)** The expression analysis of MMP15 was detected by real time PCR. *P < 0.05.

We next analyzed the CXCR4 expression in ESCC cell lines and human esophageal epithelial cell line Het-1a. As expected, we got the same result that CXCR4 was overexpressed in ESCC cell lines compared with human esophageal epithelial cell line Het-1a (Figure 3A, Supplementary Figure S1). CXCR4 expression was highest in EC109 cells and detected by flow cytometry (Figure 3B). To investigate the role of CXCR4 in ESCC, we isolated CXCR4 positive and negative cells in ESCC cell line EC109 by MACS. The purity of the two sorted subpopulations was 92.3 and 94.4%, respectively (Figure 3C). QPCR was used to confirm the expression of CXCR4 in the two subpopulations (Figure 3D). To assess whether the CXCR4 positive cells have the features of the tumor-initiating cells, we compared the mRNA expression levels of stemness-associated markers such as NANOG, OCT4, LIN28, and SOX9 between CXCR4 positive and negative cells by quantitative real-time PCR. The high expression levels of NANOG, OCT4, LIN28, and SOX9 were observed in the CXCR4 positive cells, compared with the CXCR4 negative cells (Figure 3E). Since CSCs have been demonstrated to be more resistant to chemo- and radiation therapy and thus might contribute to drug resistance and tumor recurrence, we analyzed the sensitivity of CXCR4 positive and negative cells toward cisplatin (DDP) and docetaxel (TXT). The survival rates of CXCR4 positive cells were higher under the treatment of DDP and TXT, compared with CXCR4 negative cells (Figure 3F). Further, we analyzed the migration and invasion ability in the two subpopulations. CXCR4 positive cells showed stronger migration and invasion ability (Figure 4A). AMD3100, a CXCR4 antagonist, significantly inhibit migration and invasion ability of CXCR4 positive cells (Figure 4B). And CXCR4 positive cells increased expression of MMP9 and MMP15 (Figures 4C,D), which are known to play an important role in extracellular matrix remodeling during the process of tumor invasion and metastasis. These data suggested that CXCR4 had a functional role in the maintenance of stemness of ESCC cells.

### CQ Targeted CXCR4-Positive ESCC Cells via STAT3

**Figure 5:**
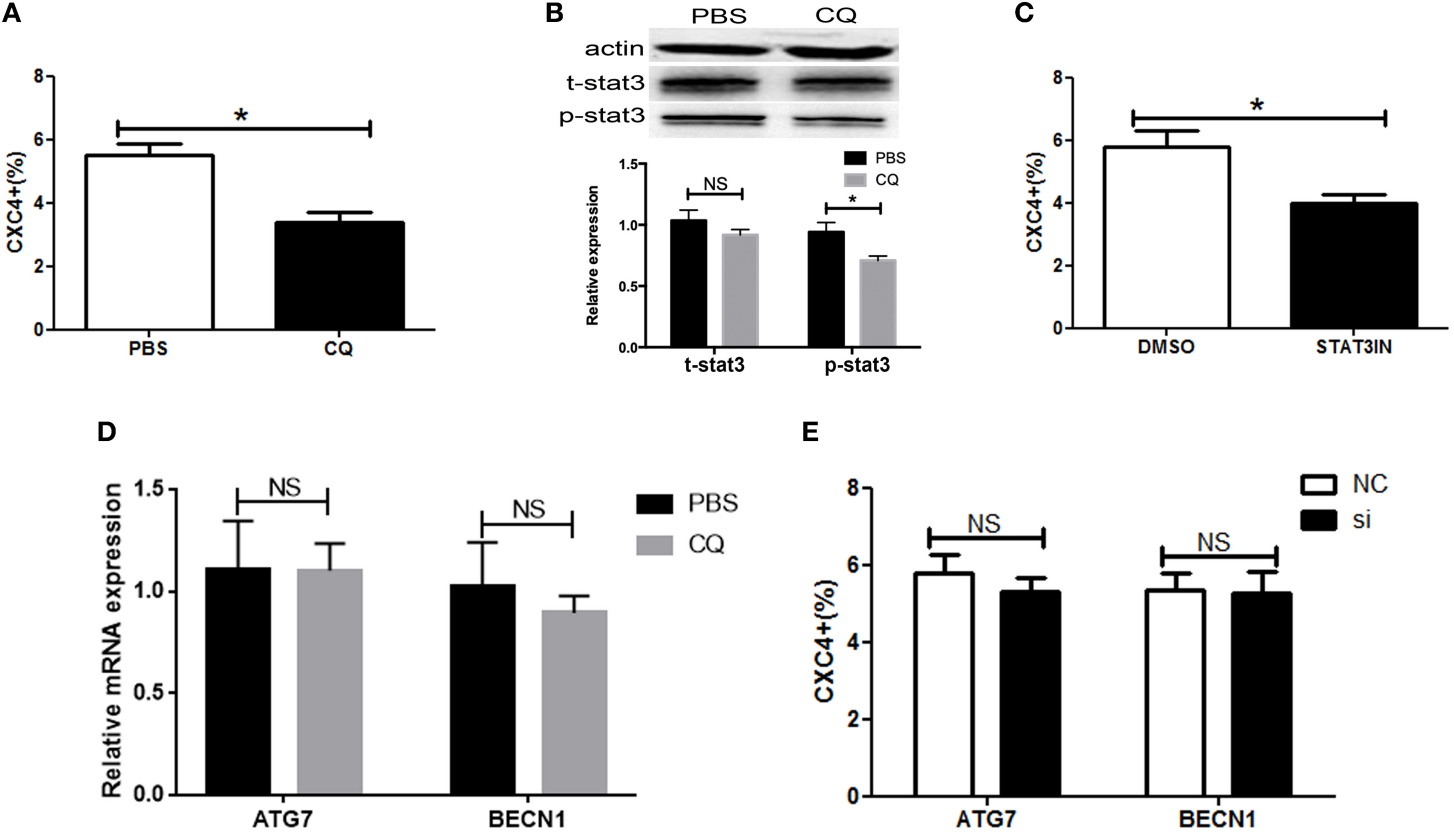
Chloroquine targeted CXCR4-positive ESCC cells via STAT3. **(A)** The expression of CXCR4 was analyzed by flow cytometry. **(B)** The activity of pSTAT3 and tSTAT3 were measured by western blotting in ESCC cells with or without CQ treatment (5 μM). **(C)** The expression of CXCR4 was analyzed by flow cytometry in ESCC cells with or without STAT3 inhibitor (S3I-201, Sigma, USA) treatment. **(D)** The expression analysis of ATG7 and BECN1 was detected by real time PCR in ESCC cells with or without CQ treatment (5 μM). **(E)** The expression of CXCR4 was analyzed by flow cytometry in ESCC cells after ATG7 or BECN1 knockdown. *P < 0.05.

Recently, CQ had been demonstrated to function as an inhibitor of CXCR4 signaling and have an impact on the cancer stem-like phenotype (20, 21, 23, 24). We aimed to assess the effect of CQ on CXCR4 expression in ESCC cells. We found CQ could reduce the amount of cells with CXCR4 expression (Figure 5A). Next, we dissected the molecular mechanisms by which CQ inhibited CXCR4 expression. Signal transducer and activator of transcription 3 (STAT3) plays a key role in the tumorigenesis and cancer stem cells (25, 26). We observed that CQ inhibited STAT3 in ESCC cells (Figure 5B). Further, we found that CXCR4 expression was decreased by the STAT3 inhibitor (Figure 5C). CQ is an anti-malarial drug known to inhibit autophagy by disrupting lysosomal stability and function. We found that CQ had no effect on the expression of ATG7 and BECN1, known as key molecules in autophagy pathway (Figure 5D). Autophagy inhibition with ATG7 or BECN1 siRNAs did not affect CXCR4 expression (Figure 5E). These data suggested that CQ targeted CXCR4-positive ESCC cells via STAT3 pathway, independent of autophagy.

### CQ Inhibited Tumor Growth *in vivo*

**Figure 6:**
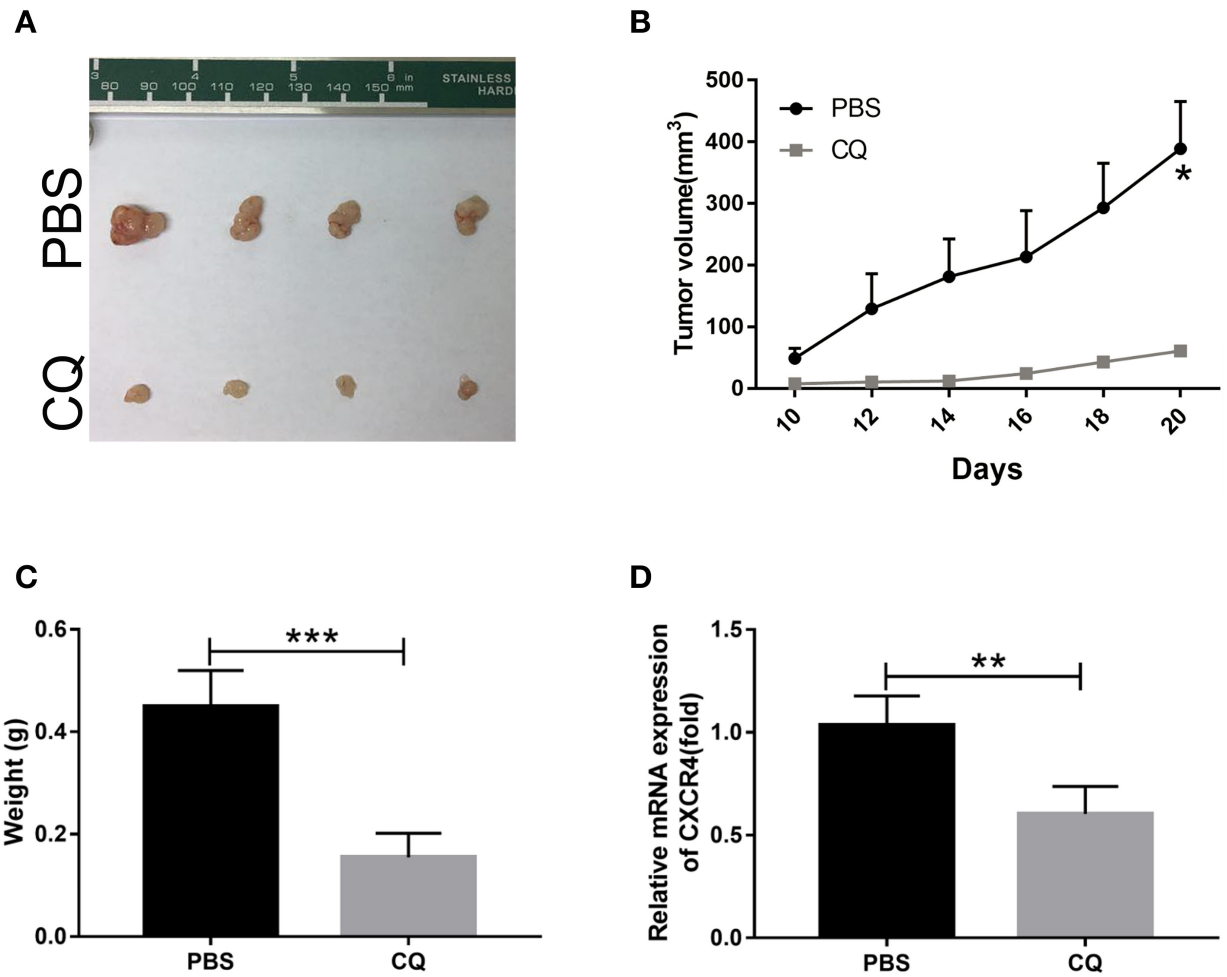
Chloroquine inhibited tumor growth *in vivo*. **(A)** Images of the xenograft tumors formed in nude mice. **(B)** Tumor volume was measured every second day and expressed in mm^3^. **(C)** Weights of xenograft tumors were summarized. **(D)** The expression of CXCR4 was confirmed. *P < 0.05, **P < 0.01, and ***P < 0.001.

The most important property of CSCs is their ability to efficiently propagate tumors *in vivo* that recapitulate the parental tumors. To examine the role of CQ on the tumor initiating capability, BALB/c nude mice were transplanted with ESCC EC109 cells and treated with or without CQ. We found that animals with CQ treatment markedly delayed the tumor progression relative to the control group (Figures 6A,B). CXCR4 and stemness-related transcription factors showed a significant decrease in animals with CQ treatment (Figures 6C,D). Taken together, these data demonstrate that CQ inhibits the tumorigenic capacity of ESCC cells *in vivo*.

## Discussion

CXCR4, also known as cluster of differentiation 184 (CD184) or fusin, one of the most well-studied chemokine receptors due to its earlier found role as a coreceptor for HIV entry (8), is widely expressed throughout the human body during embryonic development and adult life, with uniquely high-expression levels in the hematopoietic system (27). Recent studies confirmed a wide expression of CXCR4 in the tumors with a consequent implication of this receptor in a panel of pathological processes such as cell proliferation, migration, metastasis and cancer emergence and progression. Importantly, the CXCR4 has emerged as a drug target for its crucial role in promoting and maintaining cancer stem cells (CSCs). Kimura et al. found CXCR4 was associated with cell proliferation and tumorigenicity, concluding that CXCR4 is the marker of synovial sarcoma-initiating cells, a new biomarker for prognosis and a new potential therapeutic target (28). Sun et al. demonstrated that CD133^+^CXCR4^+^ primary endometrial cancer cells grew faster, exhibited high expression of stemness-related genes, produced more spheres, had higher clonogenic ability, and more resistant to anti-cancer drugs than other subpopulations, indicating that CD133^+^CXCR4^+^ cells may possess some characteristics of CSCs in primary endometrial cancer (29). CXCR4 increases the growth and sphere formation efficiency of hypoxic breast cancer side population (SP) cells by c-Jun/ABCG2 pathway (30).

In human esophageal cancer, CXCR4 overexpression promoted cell invasion *in vitro* and tumor growth *in vivo* and indicated worse survival outcome (31–33). Consistent with previous studies, we found that CXCR4 was significantly up-regulated in ESCC tissues, and correlated with tumor invasion and survival. Increasing evidence suggests that CXCR4 is potentially the CSCs marker of malignant tumors including synovial sarcoma (28), endometrial cancer (29), lung cancer (34), and so on. To investigate the role of CXCR4 in ESCC, we isolated CXCR4 positive and negative cells by MACS. We showed that CXCR4 positive cells overexpressed stem-related genes, more resistant to chemotherapy, and possessed stronger invasion ability, suggesting CXCR4 positive cells displayed stem-like properties.

Recently, the CXCL12/CXCR4 signaling pathway has emerged as a potential therapeutic target for human tumors because of its critical role in promoting and maintaining CSCs (35). Over the past few years, a number of inhibitors of CXCL12/CXCR4 have been identified and currently are in different development stages as potential agents for the treatment of cancers. So far, CXCR4 antagonists are developed by a number of programs, including five major classes: (1) small modified peptides, such as T140, (2) small-molecules, such as AMD3100, (3) antibodies, such as MDX-1338/BMS 93656, (4) modified agonists and antagonists for CXCL12 such as CTCE-9908, and (5) microRNAs, such as miR-302a (35).

CQ, a well-known antimalarial, has been administered to patients for over 60 years and the safety profiles are well-established. In malaria infection, CQ inhibits heme polymerase activity in Plasmodium parasites, thereby leading to the buildup of free heme, a substance toxic to the parasite (36). CQ has recently been reported to inhibit self-renewal of CSCs and essentially abrogate their *in-vivo* tumorigenicity. Application of CQ for cancer treatment is an example of drug re-purposing. Sotelo et al. observed that addition of CQ to standard therapy leads to a significant prolongation of survival in patients with glioblastoma (37). Until recently, the generally accepted antitumor mechanisms of CQ was inhibition of autophagy (38). Nearly simultaneously, it has been demonstrated that tumor-suppressing effects of CQ are independent from its autophagy-inhibiting activities (39, 40). Choi et al. demonstrated that CQ regulates the CSCs in triple negative breast cancer through altering Jak2-STAT3 signaling pathway and DNMT1 expression in addition to autophagy inhibition (24). It was reported that CQ targeted pancreatic cancer stem cells via inhibition of CXCR4 and hedgehog signaling, and its inhibitory effect was not related to inhibition of autophagy (21). We observed that CQ targeted CXCR4-positive ESCC cells, independent of autophagy. This is in line with recent data that CQ can act independently of its effect on autophagy.

In summary, our study demonstrates that CXCR4 is involved in the maintenance of stemness of ESCC cells and plays an oncogenic role in human ESCC. CQ targets CXCR4-positive ESCC cells via STAT3 pathway, independent of autophagy. Therefore, CQ with anti-CSCs effects, warrants further clinical evaluation as a targeted therapy, especially in combination therapy with chemotherapy, for the comprehensive treatment of ESCC.

## Data Availability Statement

All datasets generated for this study are included in the article/Supplementary Material.

## Ethics Statement

The studies involving human participants were reviewed and approved by the First Affiliated Hospital of Zhengzhou University Research Ethics Committee. The patients/participants provided their written informed consent to participate in this study. This animal study was reviewed and approved by the Animal Care and Use Committee of the First Affiliated Hospital of Zhengzhou University. Written informed consent was obtained from the individual(s) for the publication of any potentially identifiable images or data included in this article.

## Author Contributions

YZ and DY contributed conception and design of the study. DY, DZ, XS, SL, AL, DW, GQ, YP, YQ, and XC contributed to the experiments and data acquisition. FW, RC, SZ, and LW performed the statistical analysis. DY wrote the first draft of the manuscript. YZ revised the manuscript. All authors contributed to manuscript revision, read, and approved the submitted version.

### Conflict of Interest

The authors declare that the research was conducted in the absence of any commercial or financial relationships that could be construed as a potential conflict of interest.

## Supplementary Material

Supplementary Figure S1 The CXCR4 expression was analyzed by flow cytometer. Representative flow image is presented.

## References

[1] Torre LA Bray F Siegel RL Ferlay J Lortet-Tieulent J Jemal A. Global cancer statistics, 2012. CA Cancer J Clin. (2015) 65:87–108. 10.3322/caac.21262 25651787

[2] Jemal A Bray F Center MM Ferlay J Ward E Forman D. Global cancer statistics. CA Cancer J Clin. (2011) 61:69–90. 10.3322/caac.20107 21296855

[3] Lin EW Karakasheva TA Hicks PD Bass AJ Rustgi AK. The tumor microenvironment in esophageal cancer. Oncogene. (2016). 10.1038/onc.2016.34 26923327 PMC5003768

[4] Zeng H Zheng R Zhang S Zuo T Xia C Zou X. Esophageal cancer statistics in China, 2011: estimates based on 177 cancer registries. Thorac Cancer. (2016) 7:232–7. 10.1111/1759-7714.12322 27042227 PMC4773307

[5] Qiao Y Zhang C Li A Wang D Luo Z Ping Y. IL6 derived from cancer-associated fibroblasts promotes chemoresistance via CXCR7 in esophageal squamous cell carcinoma. Oncogene. (2017). 10.1038/onc.2017.387 29059160

[6] Alison MR Lin WR Lim SM Nicholson LJ. Cancer stem cells: in the line of fire. Cancer Treat Rev. (2012) 38:589–98. 10.1016/j.ctrv.2012.03.003 22469558

[7] Endres MJ Clapham PR Marsh M Ahuja M Turner JD McKnight A. CD4-independent infection by HIV-2 is mediated by fusin/CXCR4. Cell. (1996) 87:745–56. 10.1016/S0092-8674(00)81393-8 8929542

[8] Feng Y Broder CC Kennedy PE Berger EA. HIV-1 entry cofactor: functional cDNA cloning of a seven-transmembrane, G protein-coupled receptor. Science. (1996) 272:872–7. 10.1126/science.272.5263.872 8629022

[9] Bleul CC Farzan M Choe H Parolin C Clark-Lewis I Sodroski J. The lymphocyte chemoattractant SDF-1 is a ligand for LESTR/fusin and blocks HIV-1 entry. Nature. (1996) 382:829–33. 10.1038/382829a0 8752280

[10] Yang P Liang SX Huang WH Zhang HW Li XL Xie LH. Aberrant expression of CXCR4 significantly contributes to metastasis and predicts poor clinical outcome in breast cancer. Curr Mol Med. (2014) 14:174–84. 10.2174/1566524013666131121115656 24256053

[11] Sun X Charbonneau C Wei L Yang W Chen Q Terek RM. CXCR4-targeted therapy inhibits VEGF expression and chondrosarcoma angiogenesis and metastasis. Mol Cancer Ther. (2013) 12:1163–70. 10.1158/1535-7163.MCT-12-1092 23686836 PMC3707941

[12] Sobolik T Su YJ Wells S Ayers GD Cook RS Richmond A. CXCR4 drives the metastatic phenotype in breast cancer through induction of CXCR2 and activation of MEK and PI3K pathways. Mol Biol Cell. (2014) 25:566–82. 10.1091/mbc.e13-07-0360 24403602 PMC3937084

[13] Lv B Yang X Lv S Wang L Fan K Shi R. CXCR4 signaling induced epithelial-mesenchymal transition by PI3K/AKT and ERK pathways in glioblastoma. Mol Neurobiol. (2014) 52:1263–8. 10.1007/s12035-014-8935-y 25326893

[14] Gockel I Schimanski CC Heinrich C Wehler T Frerichs K Drescher D. Expression of chemokine receptor CXCR4 in esophageal squamous cell and adenocarcinoma. BMC Cancer. (2006) 6:290. 10.1186/1471-2407-6-290 17176471 PMC1766934

[15] Gassenmaier M Chen D Buchner A Henkel L Schiemann M Mack B. CXC chemokine receptor 4 is essential for maintenance of renal cell carcinoma-initiating cells and predicts metastasis. Stem Cells. (2013) 31:1467–76. 10.1002/stem.1407 23630186

[16] Jung MJ Rho JK Kim YM Jung JE Jin YB Ko YG. Upregulation of CXCR4 is functionally crucial for maintenance of stemness in drug-resistant non-small cell lung cancer cells. Oncogene. (2013) 32:209–21. 10.1038/onc.2012.37 22370645

[17] Gatti M Pattarozzi A Bajetto A Wurth R Daga A Fiaschi P. Inhibition of CXCL12/CXCR4 autocrine/paracrine loop reduces viability of human glioblastoma stem-like cells affecting self-renewal activity. Toxicology. (2013) 314:209–20. 10.1016/j.tox.2013.10.003 24157575

[18] Yang S Wang X Contino G Liesa M Sahin E Ying H. Pancreatic cancers require autophagy for tumor growth. Genes Dev. (2011) 25:717–29. 10.1101/gad.2016111 21406549 PMC3070934

[19] Firat E Weyerbrock A Gaedicke S Grosu AL Niedermann G. Chloroquine or chloroquine-PI3K/Akt pathway inhibitor combinations strongly promote gamma-irradiation-induced cell death in primary stem-like glioma cells. PLoS ONE. (2012) 7:e47357. 10.1371/journal.pone.0047357 23091617 PMC3473017

[20] Kim J Yip ML Shen X Li H Hsin LY Labarge S. Identification of anti-malarial compounds as novel antagonists to chemokine receptor CXCR4 in pancreatic cancer cells. PLoS ONE. (2012) 7:e31004. 10.1371/journal.pone.0031004 22319600 PMC3272047

[21] Balic A Sorensen MD Trabulo SM Sainz B Jr Cioffi M. Chloroquine targets pancreatic cancer stem cells via inhibition of CXCR4 and hedgehog signaling. Mol Cancer Ther. (2014) 13:1758–71. 10.1158/1535-7163.MCT-13-0948 24785258

[22] Yue D Fan Q Chen X Li F Wang L Huang L. Epigenetic inactivation of SPINT2 is associated with tumor suppressive function in esophageal squamous cell carcinoma. Exp Cell Res. (2014) 322:149–58. 10.1016/j.yexcr.2013.11.009 24269829

[23] Yu F Li J Xie Y Sleightholm RL Oupicky D. Polymeric chloroquine as an inhibitor of cancer cell migration and experimental lung metastasis. J Control Release. (2016) 244 (Pt B):347–56. 10.1016/j.jconrel.2016.07.040 27473763 PMC5167664

[24] Choi DS Blanco E Kim YS Rodriguez AA Zhao H Huang TH. Chloroquine eliminates cancer stem cells through deregulation of Jak2 and DNMT1. Stem Cells. (2014) 32:2309–23. 10.1002/stem.1746 24809620 PMC4138251

[25] Kryczek I Lin Y Nagarsheth N Peng D Zhao L Zhao E. IL-22(+)CD4(+) T cells promote colorectal cancer stemness via STAT3 transcription factor activation and induction of the methyltransferase DOT1L. Immunity. (2014) 40:772–84. 10.1016/j.immuni.2014.03.010 24816405 PMC4032366

[26] Agarwal S Lakoma A Chen Z Hicks J Metelitsa LS Kim ES. G-CSF promotes neuroblastoma tumorigenicity and metastasis via STAT3-dependent cancer stem cell activation. Cancer Res. (2015) 75:2566–79. 10.1158/0008-5472.CAN-14-2946 25908586 PMC4470771

[27] Kircher M Herhaus P Schottelius M Buck AK Werner RA Wester HJ. CXCR4-directed theranostics in oncology and inflammation. Ann Nucl Med. (2018) 32:503–11. 10.1007/s12149-018-1290-8 30105558 PMC6182637

[28] Kimura T Wang L Tabu K Tsuda M Tanino M Maekawa A. Identification and analysis of CXCR4-positive synovial sarcoma-initiating cells. Oncogene. (2016) 35:3932–43. 10.1038/onc.2015.461 26640147

[29] Sun Y Yoshida T Okabe M Zhou K Wang F Soko C. Isolation of stem-like cancer cells in primary endometrial cancer using cell surface markers CD133 and CXCR4. Transl Oncol. (2017) 10:976–87. 10.1016/j.tranon.2017.07.007 29096246 PMC5671417

[30] He C Zhang H Wang B He J Ge G. SDF-1/CXCR4 axis promotes the growth and sphere formation of hypoxic breast cancer SP cells by c-Jun/ABCG2 pathway. Biochem Biophys Res Commun. (2018) 505:593–99. 10.1016/j.bbrc.2018.09.130 30274780

[31] Guo J Yu X Gu J Lin Z Zhao G Xu F. Regulation of CXCR4/AKT-signaling-induced cell invasion and tumor metastasis by RhoA, Rac-1, and Cdc42 in human esophageal cancer. Tumour Biol. (2016) 37:6371–8. 10.1007/s13277-015-4504-x 26631033

[32] Wu J Wu X Liang W Chen C Zheng L An H. Clinicopathological and prognostic significance of chemokine receptor CXCR4 overexpression in patients with esophageal cancer: a meta-analysis. Tumour Biol. (2014) 35:3709–15. 10.1007/s13277-013-1490-8 24326770

[33] Lu CL Guo J Gu J Ge D Hou YY Lin ZW. CXCR4 heterogeneous expression in esophageal squamous cell cancer and stronger metastatic potential with CXCR4-positive cancer cells. Dis Esophagus. (2014) 27:294–302. 10.1111/dote.12100 23822165

[34] Chen WL Huang AF Huang SM Ho CL Chang YL Chan JY. CD164 promotes lung tumor-initiating cells with stem cell activity and determines tumor growth and drug resistance via Akt/mTOR signaling. Oncotarget. (2017) 8:54115–35. 10.18632/oncotarget.11132 28903328 PMC5589567

[35] Zhou W Guo S Liu M Burow ME Wang G. Targeting CXCL12/CXCR4 axis in tumor immunotherapy. Curr Med Chem. (2017) 26:3026–41. 10.2174/0929867324666170830111531 28875842 PMC5949083

[36] Pelt J Busatto S Ferrari M Thompson EA Mody K Wolfram J. Chloroquine and nanoparticle drug delivery: a promising combination. Pharmacol Ther. (2018) 191:43–9. 10.1016/j.pharmthera.2018.06.007 29932886 PMC6677248

[37] Sotelo J Briceno E Lopez-Gonzalez MA. Adding chloroquine to conventional treatment for glioblastoma multiforme: a randomized, double-blind, placebo-controlled trial. Ann Int Med. (2006) 144:337–43. 10.7326/0003-4819-144-5-200603070-00008 16520474

[38] Cai Y Cai J Ma Q Xu Y Zou J Xu L. Chloroquine affects autophagy to achieve an anticancer effect in EC109 esophageal carcinoma cells *in vitro*. Oncol Lett. (2018) 15:1143–8. 10.3892/ol.2017.7415 29422973 PMC5772993

[39] Eng CH Wang Z Tkach D Toral-Barza L Ugwonali S Liu S. Macroautophagy is dispensable for growth of KRAS mutant tumors and chloroquine efficacy. Proc Natl Acad Sci USA. (2016) 113:182–7. 10.1073/pnas.1515617113 26677873 PMC4711870

[40] King MA Ganley IG Flemington V. Inhibition of cholesterol metabolism underlies synergy between mTOR pathway inhibition and chloroquine in bladder cancer cells. Oncogene. (2016) 35:4518–28. 10.1038/onc.2015.511 26853465 PMC5000518

