# Corrigendum: Chloroquine Inhibits Stemness of Esophageal Squamous Cell Carcinoma Cells Through Targeting CXCR4-STAT3 Pathway

**DOI:** 10.3389/fonc.2022.860158

**Published:** 2022-02-24

**Authors:** Dongli Yue, Daiqun Zhang, Xiaojuan Shi, Shasha Liu, Anqi Li, Dong Wang, Guohui Qin, Yu Ping, Yamin Qiao, Xinfeng Chen, Feng Wang, Renyin Chen, Song Zhao, Lidong Wang, Yi Zhang

**Affiliations:** ^1^Biotherapy Center, The First Affiliated Hospital of Zhengzhou University, Zhengzhou, China; ^2^Cancer Center, The First Affiliated Hospital of Zhengzhou University, Zhengzhou, China; ^3^Department of Pathology, The First Affiliated Hospital of Zhengzhou University, Zhengzhou, China; ^4^Department of Thoracic Surgery, The First Affiliated Hospital of Zhengzhou University, Zhengzhou, China; ^5^Henan Key Laboratory for Esophageal Cancer Research and State Key Laboratory for Esophageal Cancer Prevention & Treatment of The First Affiliated Hospital of Zhengzhou University, Zhengzhou, China; ^6^School of Life Sciences, Zhengzhou University, Zhengzhou, China; ^7^Henan Key Laboratory for Tumor Immunology and Biotherapy, Zhengzhou, China

**Keywords:** esophageal squamous cell carcinoma (ESCC), cancer stem cells (CSCs), CXCR4, chloroquine (CQ), STAT3

In the original article, there was a mistake in **Figures 3C** and **5B** as published. In **Figure 3C**, the representative image of the purity of the two sorted CXCR4 positive/negative subpopulations was inadvertently presented with an incorrect picture. In **Figure 5B**, incorrect image of western result of t-stat3 and p-stat3 were imported by mistake. The corrected **Figures 3** and **5** appears below.

**Figure 3:**
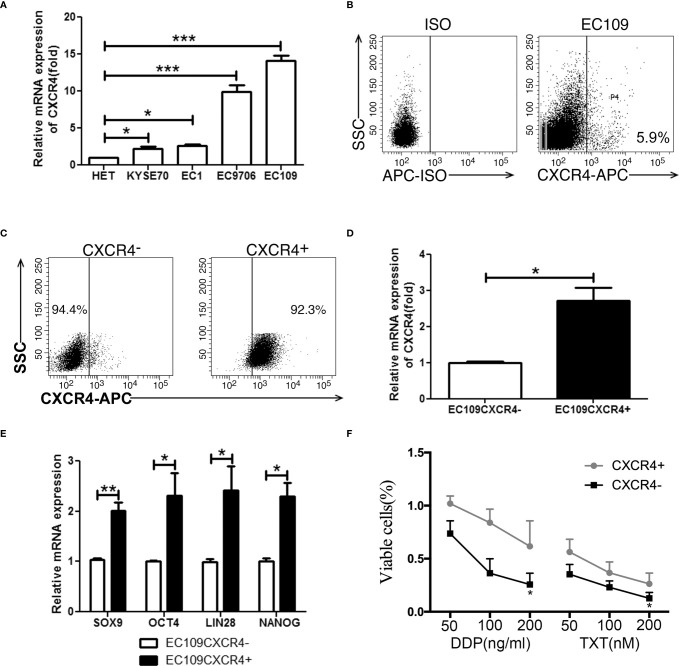
CXCR4 positive cells possessed stem-like properties. **(A)** The CXCR4 expression was detected by real time PCR in 1 immortalized esophageal cell line (Het-1a) and 4 ESCC cell lines. **(B)** The expression analysis of CXCR4 was detected by flow cytometry. **(C)** The purity of sorted EC109 cells with or without CXCR4 expression. **(D)** The expressio analysis of CXCR4 was detected by real time PCR. **(E)** The expression analysis of stemness-related transcription factors (SOX9, OCT4, LIN28, and NANOG) was detected by real time PCR. **(F)** Cell survival rate was tested by CCK-8 method. *P < 0.05, **P < 0.01, and ***P < 0.001.

**Figure 5:**
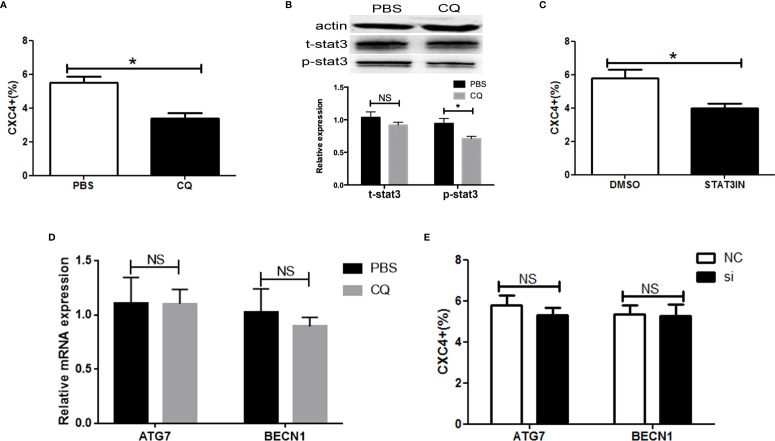
Chloroquine targeted CXCR4-positive ESCC cells via STAT3. **(A)** The expression of CXCR4 was analyzed by flow cytometry. **(B)** The activity of pSTAT3 and tSTAT3 were measured by western blotting in ESCC cells with or without CQ treatment (5µM). **(C)** The expression of CXCR4 was analyzed by flow cytometry in ESCC cells with or without STAT3 inhibitor (S3I-201, Sigma, USA) treatment. **(D)** The expression analysis of ATG7 and BECN1 was detected by real time PCR in ESCC cells with or without CQ treatment (5µM). **(E)** The expression of CXCR4 was analyzed by flow cytometry in ESCC cells after ATG7 or BECN1 knockdown. *P < 0.05.

The authors apologize for these errors and state that this does not change the scientific conclusions of the article in any way. The original article has been updated.

## Publisher’s Note

All claims expressed in this article are solely those of the authors and do not necessarily represent those of their affiliated organizations, or those of the publisher, the editors and the reviewers. Any product that may be evaluated in this article, or claim that may be made by its manufacturer, is not guaranteed or endorsed by the publisher.

