# Supplementary figures and images for: Chloroquine Inhibits Stemness of Esophageal Squamous Cell Carcinoma Cells Through Targeting CXCR4-STAT3 Pathway
Source: Front Oncol. 2020 Mar 13;10:311. doi: 10.3389/fonc.2020.00311 (PMC7083143; doi:10.3389/fonc.2020.00311)

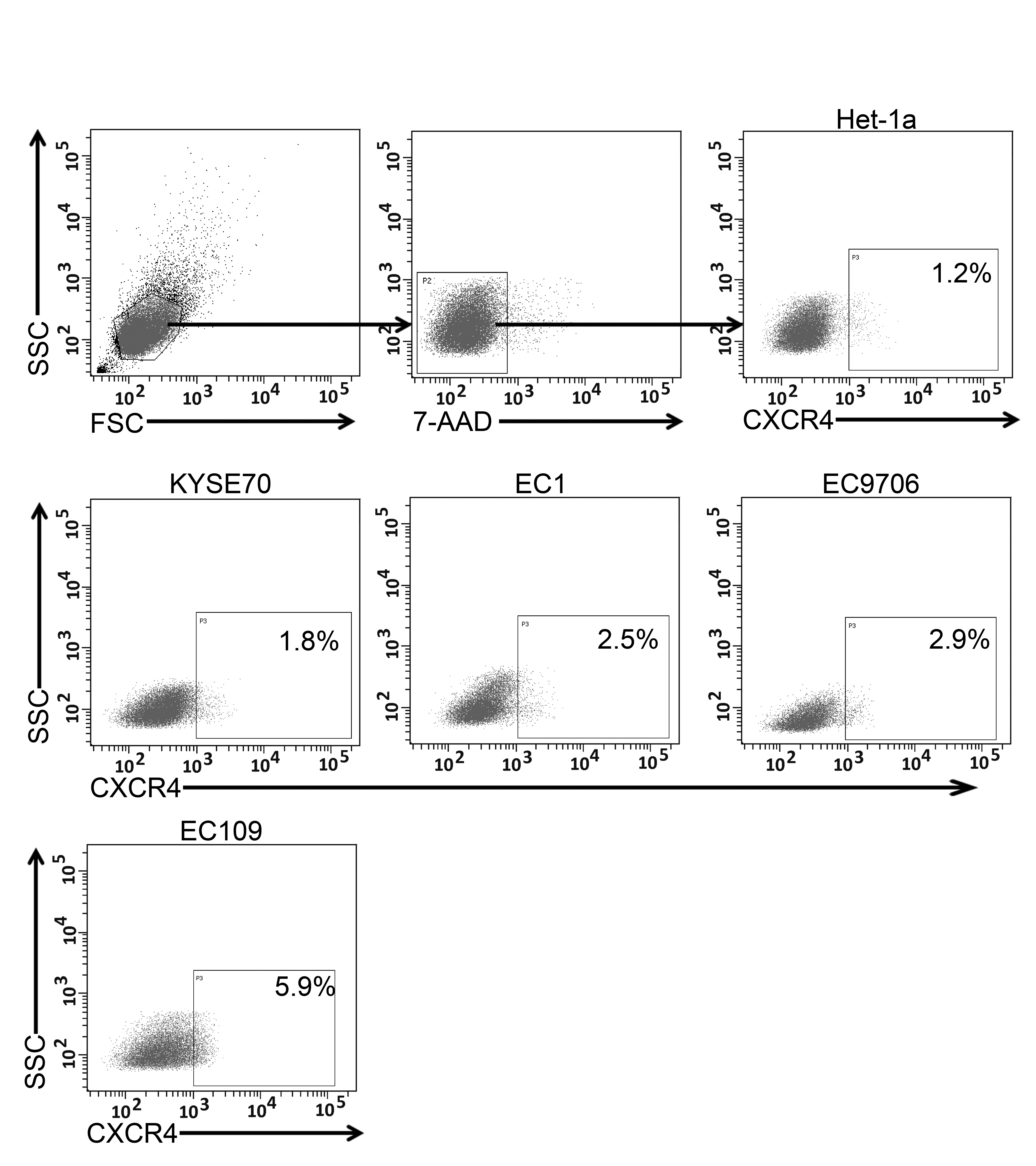

Supplement: Supplementary Figure S1 — The CXCR4 expression was analyzed by flow cytometer. Representative flow image is presented. [file Image_1.TIF]
